# Pharmaceutical Processes

**Published:** 1800-02

**Authors:** 


### *Pharmaceutical Proceſſes.*

Profeſſor Hufeland, of Jena, in his Journal of the Practice of Medicine, Vol. IV. Number II p. 360, mentions the *Sulphat of Ammonia*, as a remedy much brought into practice in England. Prof. Tromsdorf recommends the following method of preparing it, which is worthy of attention. Liver of ſulphur produced by melting equal parts of ſulphur and potaſs, is powdered, placed in a phial, and the ſulphureo-hydrogen gas being diſengaged by means of diluted muriatic acid, is tranſmitted into a high jar, filled with *ſpiritus ſalis ammoniaci*, which has been obtained by diſtilling one part from a mixture of one part of ſal-ammoniac, one fourth part of powdered quick lime, and ſix parts of water. The ſulphureo-hydrogen gas combines more readily with the liquid ammoniac, in proportion to its coldneſs. It muſt be diſengaged till no more can be abſorbed by the ammonia. The preparation requires to be well defended from air in a cloſely ſtopped glaſs veſſel.—*Journ. der Pharmacie*, B. v. p. 149.

---

### *On the ſeparation of Soda from Marine Salt, and the Depuration of Potaſs.*

A mixture of twenty parts of muriat of ſoda and thirteen parts of the carbonat of potaſs, produces muriat of potaſs, and carbonat of ſoda; but the two ſalts are ſeparated with great difficulty. Cit. Van Mons has, however, adopted a very proper and effectual method. He deprives the ſoda of its carbonic acid by quick lime; the ſoda thus rendered cauſtic, remains in the mother ley uncryſtallized, while the muriat of potaſs forms cryſtals. The ſoda muſt afterwards be evaporated, and ignited in a crucible with powdered charcoal. Thus it again becomes mild, ſuſceptible of cryſtallization, and may be eaſily purified.

The depuration of potaſs may be performed in a manner exactly ſimilar to the preceding, by which it may be purified of all its heterogeneous ingredients.—*Chem. Annal.* B. I. p. 40.

---

### *Vitriolated Kali.*

M. Kasteleyn added twenty-four parts of ſulphat of magneſia to twenty parts of muriat of potaſs. He obtained muriat of magneſia, and ſulphat of potaſs. The former being very deliqueſcent, the latter is eaſily ſeparated by cryſtallization. The product is from ſixteen to eighteen parts of vitriolated tartar, and twelve parts of a dry muriat of magneſia.—*Ibid.* p. 41.

### *Magnesia.*

The muriat of magnesia, which was produced during the preparation of vitriolated kali from muriat of potass and sulphat of magnesia, requires only to be distilled in a retort, with some water at a proper temperature, till no vapours arise, in order to deprive it of all its muriatic acid, which is but slightly combined with the magnesia. The produce is two-thirds in weight of the employed muriat of magnesia, and is tolerably concentrated. The third part, which remains in the retort, is pure magnesia, and which, edulcorated in water, may be deprived of the small quantity of a neutral salt which still adheres to it undecomposed. *Ibid.* p. 41.

---

### *Muriat of Barytes.*

Dr. RICHTER employs an excellent method of exhibiting this salt in pure white crystals, without expence or loss of time. He mixes pulverized heavy spar into a paste, with a saturated solution of carbonat of potass, and suffers it to dry as quickly as possible; he then pounds it again, and subjects it to a red heat. The vitriolated kali thus obtained, is edulcorated with water, and the earthy residuum treated with muriatic acid in the usual manner. The remaining undissolved ponderous spar, is afterwards moistened with a solution of potass, dried, and ignited. This process is repeated till all the spar is dissolved, or till there remains but a very inconsiderable quantity. The muriatic solutions of barytes are afterwards evaporated to dryness, and the inspissated ley is melted in a crucible, poured out on a tin plate, dissolved in five times its weight of water, and purified by filtration. The liquor thus treated, is transparent like water, and when slowly evaporated and refrigerated, yields the purest and most regular crystals of barytes: the crystallization is continued till there is an alteration observed in the form of the crystals, which indicates the appearance of the strontian earth that is contained in the ponderous spar. By this method, a much smaller quantity of potass is required than in the usual mode of preparation, by melting potass together with ponderous spar. *Ibid.* B. I. p. 334.

---

### *An improved Method of preparing Plasters and Ointments, in which fresh Plants or their Juices are used as Ingredients; such as Empl. Cicutæ, Ungu. Popul. &c.*

Citizen VAN MONS carefully expresses the juice of plants, and exposes it to a moderate heat. The extract thus produced, is is dried as much as it will admit of, pounded in a mortar, and put on the fire with oleaginous ingredients, till the evaporation of all humidity is effected: by this means, he introduces into the plasters, only those parts of the plants which are soluble in fat substances, and avoids the tedious evaporation which decomposes the most efficacious vegetable particles. This preparation has, besides, this advantage, that its proportion can be more exactly ascertained, as it is deprived of all heterogeneous matter, which is often combined with the common extracts. *Ibid.* p. 636.

### *Concentrated Lemon Juice.*

Dr. Brugnatelli filters the expressed juice of ripe lemons through linen. He then adds some highly rectified alcohol, and places the mixture for some days in a closely stopped phial; during this time, a considerable quantity of mucous sediment is deposited, which must be separated by passing the liquid through a filtering paper; and if it should be too thick for percolation, it ought to be mixed with an additional quantity of alcohol. This filtrated liquor contains the purest citric acid, combined with alcohol; the latter may be separated by slow evaporation. After having been deprived of all the alcohol and watery particles, the acid assumes a yellowish colour, and is so strong, as to be similar in taste to a mineral acid. *Ibid.* B. I. p. 160.

### *Hahnemann's Liquor probatorius fortior.*

Two drachms of the essential acid of tartar are dissoved in sixteen ounces of pure water, at a temperature of 90°; to this solution are added two drachms of hepar sulphuris, reduced to a fine powder. The mixture is shaken for ten minutes in a capacious bottle, and then allowed to stand quietly for half an hour. The clear fluid must be decanted into another bottle, containing additional four drachms of the essential acid of tartar, which is easily dissolved by shaking it; if the liquor should be turbid, it ought to stand for twenty-four hours, and should afterwards be poured off slowly into another vessel.

This acidulated hepatic solution is of a milky colour; and a table-spoonful of it poured on two or three ounces of a suspected wine, which is adulterated with sugar of lead, or other metallic ingredients, will form a precipitate of a more or less brown colour; but if the wine remain limpid, we may conclude that it is pure and free at least from metallic adulteration. *Göttllings praktische Vortheile für Apotheker. Ed. 3d.* p. 353.

